# Assessing the impacts of recreation on the spatial and temporal activity of mammals in an isolated alpine protected area

**DOI:** 10.1002/ece3.10733

**Published:** 2023-11-28

**Authors:** Mitchell J. E. Fennell, Adam T. Ford, Tara G. Martin, A. Cole Burton

**Affiliations:** ^1^ Faculty of Forestry University of British Columbia Vancouver British Columbia Canada; ^2^ Irving K Barber Faculty of Science University of British Columbia Okanagan Kelowna British Columbia Canada; ^3^ Biodiversity Research Centre University of British Columbia Vancouver British Columbia Canada

**Keywords:** camera traps, human impact, human‐wildlife coexistence, mammal conservation, park effectiveness, recreation ecology

## Abstract

The management objectives of many protected areas must meet the dual mandates of protecting biodiversity while providing recreational opportunities. It is difficult to balance these mandates because it takes considerable effort to monitor both the status of biodiversity and impacts of recreation. Using detections from 45 camera traps deployed between July 2019 and September 2021, we assessed the potential impacts of recreation on spatial and temporal activity for 8 medium‐ and large‐bodied terrestrial mammals in an isolated alpine protected area: Cathedral Provincial Park, British Columbia, Canada. We hypothesised that some wildlife perceive a level of threat from people, such that they avoid ‘risky times’ or ‘risky places’ associated with human activity. Other species may benefit from associating with people, be it through access to anthropogenic resource subsidies or filtering of competitors/predators that are more human‐averse (i.e., human shield hypothesis). Specifically, we predicted that large carnivores would show the greatest segregation from people while mesocarnivores and ungulates would associate spatially with people. We found spatial co‐occurrence between ungulates and recreation, consistent with the human shield hypothesis, but did not see the predicted negative relationship between larger carnivores and humans, except for coyotes (*Canis latrans*). Temporally, all species other than cougars (*Puma concolor*) had diel activity patterns significantly different from that of recreationists, suggesting potential displacement in the temporal niche. Wolves (*Canis lupus*) and mountain goats (*Oreamnos americanus*) showed shifts in temporal activity away from people on recreation trails relative to off‐trail areas, providing further evidence of potential displacement. Our results highlight the importance of monitoring spatial and temporal interactions between recreation activities and wildlife communities, in order to ensure the effectiveness of protected areas in an era of increasing human impacts.

## Graphical abstract

Protected areas have a dual mandate of providing recreational activities while also protecting wildlife and their habitats. We looked at the effects of human recreation on wildlife use of habitat in space and time, and found responses to recreation varied by species. Within one protected area, we saw that animals may be avoiding humans by shifting their use of habitat in time, more than in space.

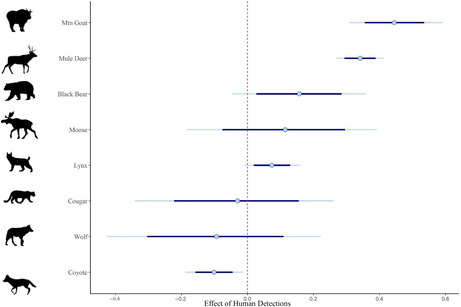

## 1 INTRODUCTION

The creation of protected areas (e.g., parks, nature reserves) is a broad conservation strategy used to try to mitigate the effects of anthropogenic stressors on wildlife (Graham et al., 2019; Haight & Hammill, 2020). Most protected areas prevent extensive land‐use change within their boundaries, providing vital habitat for wildlife in the face of an expanding human footprint (Geldmann et al., 2013; Heino et al., 2015). These areas may provide refugia from climate change effects, though connectivity across networks of protected areas is crucial for these refugia to be effective (Graham et al., 2019; Haight & Hammill, 2020; Stralberg et al., 2020). In addition to these broad‐scale protections, protected areas also provide an opportunity for increased regulation of human behaviours when compared to unprotected lands, allowing for more effective conservation actions relating to human activity.

Protected areas are often created with the dual mandate of protecting wildlife and providing opportunities for outdoor recreation and nature‐based tourism. The first of these mandates, protecting wildlife and the habitats in which they live (Pringle, 2017; Watson et al., 2014), has been broadly successful in many areas (Chen et al., 2022). However, many of these same areas are increasingly managed to provide outdoor recreational opportunities for people (Balmford et al., 2009; Reed & Merenlender, 2008). Engagement with nature positively influences human well‐being and is considered a cultural ecosystem service frequently provided by protected areas (Willis, 2015). Parks and reserves can also provide economic benefits from tourism, which in turn support conservation within these areas (Naidoo et al., 2019; Wittemyer et al., 2008). While these benefits are broadly accepted, the rapid growth of recreational activities in protected areas worldwide may be increasing the risk of negative impacts on the wildlife these areas are mandated to protect (Balmford et al., 2015; Margules & Pressey, 2000; Pressey et al., 2015).

With this dual mandate, protected area management frequently involves a difficult balance between conservation actions and access for recreationists (Whittington et al., 2019). As such, linking science to decision‐making is a key factor in effective management (Geldmann et al., 2013; Lemieux et al., 2018; Merkle et al., 2019; Pullin & Knight, 2009). Where restrictions on human activities are imposed in the absence of strong evidence of impact, they may be subject to challenge from the public, whereas unrestricted use without monitoring may jeopardise conservation values. Scientists must consider the applicability of their research to informing management decisions in a conservation context, particularly within a framework of adaptive management (Tanner‐McAllister et al., 2017). The iterative process of developing and testing hypotheses alongside management is crucial to adaptive management, ensuring recreation and conservation can be compatible in protected areas.

Under the founding doctrines of the ‘North American Model’, wildlife management and its related sciences have focused on the consumptive use of vertebrate wildlife (i.e., hunting and fishing; Hessami et al., 2021). The historical focus on hunting has meant that the science of ‘recreation ecology’ is relatively underserved, particularly in protected areas where hunting is often prohibited (Buckley, 2013; Kays et al., 2017; Marion et al., 2016). Understanding the impacts of non‐consumptive recreation on wildlife is an important consideration for effective ecosystem management within and outside of protected areas (Baas et al., 2020).

Outdoor recreation can affect wildlife and the ecosystems in which they live at multiple scales, from small‐scale spatiotemporal shifts in habitat use to broad avoidance of entire areas due to high human use (Kays et al., 2017; Larson et al., 2016; Larson et al., 2019; Naidoo & Burton, 2020). Potential costs of these shifts for wildlife may include reductions in time available for foraging (Frey et al., 2017), reduced availability of quality habitat for foraging or rearing young (Brown et al., 1999), increased risk of predation (Hamel & Côté, 2007) and chronic stress resulting in population‐level effects such as reduced survival or fecundity (Clinchy et al., 2013). Nevertheless, little quantitative information about the relationships between recreation and particular species or entire animal communities is available to inform effective management. This uncertainty can reduce trust in managers, particularly where management actions may receive substantial public opposition (Pretty & Smith, 2004), such as trail closures and quotas or temporal restrictions on human use (Whittington et al., 2019). A key factor limiting evidence‐based decisions across many protected areas is inadequate monitoring of biodiversity and visitor use (Balmford et al., 2015; Buckley, 2009).

Past studies investigating relationships between recreation and wildlife have frequently lacked direct quantification of human activity at temporal or spatial scales fine enough to test relationships with wildlife activity, thus limiting the ability to link animal responses to recreational impacts (Balmford et al., 2015; Buckley, 2009). Camera traps can provide such direct quantification of activity with minimal effect on humans or wildlife. With the growing use of this technology for studying wildlife worldwide (Burton et al., 2015; Wearn & Glover‐Kapfer, 2019) extension of camera trap sampling to directly quantify human use of the same habitats as wildlife provides opportunity for further inferences in recreational ecology (Naidoo & Burton, 2020).

Capitalising on the opportunity for ecological insight derived from camera traps, we sought to investigate the question: *does non‐consumptive human recreation displace medium‐ and large‐bodied mammals?* Specifically, we investigated whether human recreation impacts the spatiotemporal activity of medium‐ and large‐bodied mammals in Cathedral Provincial Park (hereafter Cathedral) in British Columbia, Canada. Cathedral presents a useful case study as it faces common issues of increasing human recreation, limited quantification of human use and poor understanding of the compounding effects of these factors on management efforts. Additionally, Cathedral faces increasing isolation from other high‐quality wildlife habitats due to transportation infrastructure, forestry, agriculture and urban development in the surrounding areas. Given such isolation, the potential for negative impacts of recreation on wildlife within the park takes on added significance (i.e., limited potential for population resilience or recovery due to immigration from elsewhere).

We considered two general hypotheses that could explain wildlife responses to recreation: avoidance of risk or attraction to benefits. Firstly, wildlife may perceive risk from humans, similar to the risk perceived by prey from an apex predator (Dröge et al., 2017; Suraci et al., 2019), and accordingly may avoid ‘risky places’ or ‘risky times’. For the former, wildlife may avoid areas used more frequently by humans, as predicted by the ‘landscape of fear’ hypothesis (Bleicher & Rosenzweig, 2018; Gaynor et al., 2019; Støen et al., 2015). This pattern may be shaped by previous direct persecution as well as ongoing threats from close human encounters leading to negative outcomes ranging from chronic stress to reduced fecundity (Carter & Linnell, 2016; Støen et al., 2015). Alternatively, wildlife may avoid ‘risky times’ through a temporal shift in habitat use, whereby animals become more nocturnal or crepuscular to avoid overlap with daytime human activity (Gaynor et al., 2018; Oriol‐Cotterill et al., 2015; Shamoon et al., 2018). Animals sharing space with people may be partitioning time generally, resulting in low overlap with times of greater human activity across all sites, or more selectively where risk is higher in space, such as avoiding on‐trail sites during the day (Barrueto et al., 2014). While generalist species may be able to move to other habitats due to low costs of spatial avoidance, species that are habitat specialists may depend more on temporal shifts to avoid recreation activities occurring in essential habitats (Sarmento & Berger, 2017). Ultimately, wildlife are likely to make decisions along a spatiotemporal continuum to balance the impacts of shifts in space or time with the potential costs of not reacting (Palmer et al., 2022).

Our contrasting hypothesis is that wildlife may perceive benefits from proximity to humans, such as the provision of resources or safety from predators. For prey species, human activity may repel large carnivores, providing a form of refuge under the human shield hypothesis (Muhly et al., 2011; Sarmento & Berger, 2017). Resource provision facilitated by human presence may take multiple forms, ranging from direct nutrient supplementation by humans (i.e. feeding, access to garbage and salt supplementation; Newsome et al., 2015) to indirect effects such as increasing small prey availability for mesocarnivores under the human shield hypothesis, where small prey move in when predators are repelled by humans (Muhly et al., 2011). Some species, such as mountain goats, may become dependent on humans for nutritional supplementation in the form of salt from urine or grey water disposal (Slabach et al., 2015), leading to positive spatial correlation and potential conflict. This dependence may result in an attractive sink, where costs associated with the attraction, such as direct human conflict, result in a net‐negative impact (Robinson et al., 2008). Species may also show no response to human recreation, suggesting little direct impact from current levels of recreation, with environmental variables playing a larger role in influencing habitat use.

In the context of Cathedral, we predicted that larger carnivore species (black bear; *Ursus americanus*, cougar; *Puma concolor*, grey wolf; *Canis lupus*) would avoid areas of human recreation due to the risk of persecution under a landscape of fear, either from hunting in portions of the park with less human use or destruction by managers in areas with more human use (i.e. conflict mitigation; Klees van Bommel et al., 2020). We predicted that temporal avoidance of people may occur where these species are active near infrastructure like trails and campgrounds (Table 1). Alternatively, the benefits to carnivores of easier travel on linear features such as trails may outweigh any risk associated with human encounters, leading to more spatial overlap (Dickie et al., 2017; Tattersall et al., 2020; Whittington et al., 2011). We predicted that mesocarnivore species (Canada lynx; *Lynx canadensis*, coyote; *Canis latrans*) would exhibit limited spatial avoidance of humans due to a decreased risk of direct persecution (management destruction, e.g. killing of habituated animals) as compared to larger carnivores. We also predicted that small prey of mesocarnivores (such as snowshoe hare; *Lepus americanus*) may increase in proximity to humans under the human shield hypothesis where larger predators are repelled, which may further attract mesocarnivores to areas of human use due to increased prey availability. Further, we predicted that mesocarnivores would still minimise risk of persecution by humans under a landscape of fear, resulting in increased cathemeral or nocturnal activity patterns in response to higher human activity (Frey et al., 2020; Table 1). We additionally predicted that moose (*Alces alces*), mule deer (*Odocoileus hemionus*) and mountain goats (*Oreamnos americanus*) would exhibit a positive spatiotemporal response to humans to benefit from the ‘human shield’ effect, but that habitat specialist ungulates such as mountain goats might display a temporal shift away from mid‐day due to the high overlap between key habitat and human recreation in alpine and sub‐alpine environments at that time (Table 1).

**TABLE 1:** Predicted response to human recreation for commonly encountered medium and large‐bodied mammal species in Cathedral Provincial Park. Predicted spatial and temporal responses are listed for each species, with + representing a positive response to human recreation (attraction), − a negative (avoidance) and +/− a neutral response.

Species	Predicted response		Hypothesis	Sources
	Space	Time		
Black bear *Ursus americanus*	−	−	Landscape of fear	Baker & Leberg (2018), Erb et al. (2012)
Cougar *Puma concolor*	−	−	Landscape of fear	Kays et al. (2017), Nickel et al. (2020), Reilly et al. (2017)
Grey Wolf *Canis lupus*	−	−	Landscape of fear	Kojola et al. (2016), Lesmerises et al. (2012), Musiani et al. (2010)
Canada lynx *Lynx canadensis*	+/−	−	Human shield (exploitation of increased prey due to large carnvores being repelled)	Kolbe & Squires (2007), Squires et al. (2019)
Coyote *Canis latrans*	+/−	−	Human shield (exploitation of increased prey due to large carnvores being repelled)	George & Crooks (2006), Kays et al. (2017), Nickel et al. (2020)
Moose *Alces alces*	+	+/−	Human shield	Naidoo & Burton (2020)
Mountain goat *Oreamnos americanus*	+	−	Human shield & Human resource exploitation	Festa‐Bianchet & Côté (2008), Richard & Côté (2016), Sarmento & Berger (2017)
Mule deer *Odocoileus hemionus*	+	+/−	Human shield	George & Crooks (2006), Parsons et al. (2016), Reilly et al. (2017)

*Note*: Also included is the hypothesis supporting the prediction and key associated references.

## 2 METHODS

### 2.1 Study area

**FIGURE 1:**
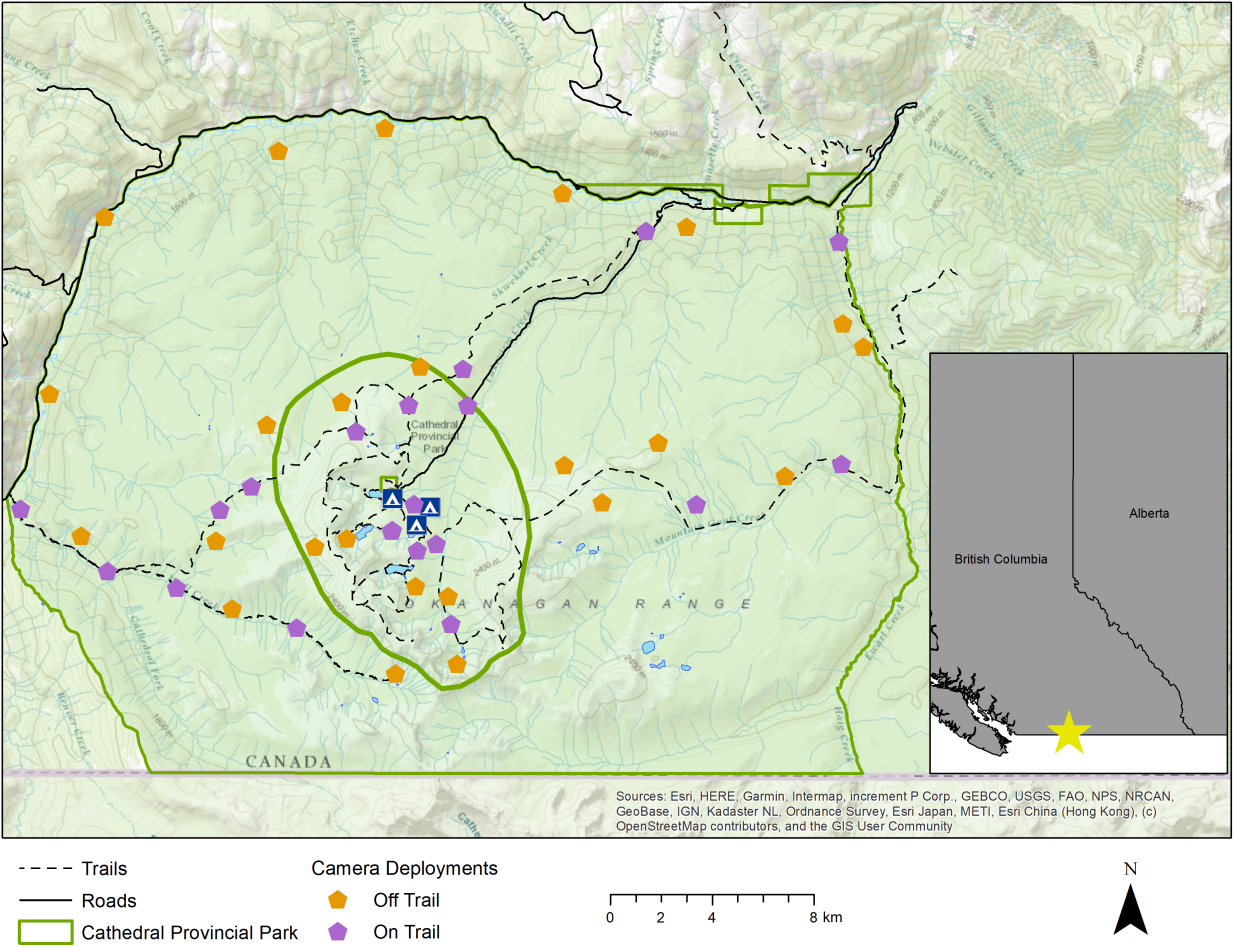
Map of camera trap deployments in Cathedral Provincial Park. The outer green polygon is the park boundary, while the inner is the park core area boundary where recreation is highest. Solid black lines are roads, and dashed lines are trails. Orange pentagons represent off‐trail camera traps, and purple pentagons represent on‐trail camera traps.

Cathedral is in southern British Columbia, near the town of Keremeos, on unceded Syilx (Okanagan) Nation territory (Figure 1). It encompasses varied vegetation types and structures across an area of 330 km^2^, and spans a large elevational gradient from 750 m to over 2600 m. The park is bounded by the Ashnola River, the Snowy Protected Area and the Canada‐United States border. Our study area extended to the park's outer boundary and included a smaller core area of approximately 50 km^2^ delineated by managers as an area of higher human use within the centre of the park (Figure 1). Biogeoclimatic zones present within the park (in decreasing order of predominance) are Engelman Spruce‐Subalpine Fir (ESSF), Montane Spruce (MS), Interior Douglas Fir (IDF) and Interior Mountain‐Heather Alpine (IMA; British Columbia Ministry of Forests, Lands and Natural Resource Operations, 2020).

The park receives over 4000 person‐days of visitation by campers annually, although tracking of total visitor use is incomplete as non‐camping visitors are not tracked (Parks, 2019). Anecdotal evidence suggests overall visitation, including day users, continues to increase year over year, based on communications with the park operator and private lodge staff (M. Fennell, *Pers. Comm*.). A small parcel of private land pre‐dating the establishment of the park is centrally located at Quiniscoe Lake in the core area and is operated as a backcountry lodge. Associated with this lodge is a private 4 × 4 access road that provides paid shuttle services for lodge guests and campers. All access to the park is by foot or the private shuttle, as mountain biking and other motorised recreation are not permitted. Hunting is prohibited within the core area of the park (Figure 1), while limited hunting for bear, deer, elk, wolf, coyote, cougar and moose occurs legally in other portions of the park from August 25 to April 15 (outside of the common recreation period) during general open seasons for each species. We observed only two independent detections of hunters in Cathedral across all cameras throughout the study period. Anecdotal evidence from land managers, business owners operating in the park and hunters encountered in the field (in areas adjacent to the park) suggests there is very limited hunting pressure within the park.

### 2.2 Camera trap survey

From July 2019 until September 2021, we operated 45 Reconyx HP2X (Reconyx, Holmen, USA) camera traps throughout much of the 330 km^2^ area of the Cathedral (Figure 1). Camera sites were selected using a stratified random design to sample across a gradient of expected recreation activity, including on and off of recreation trails and in and out of the core area (in which trail density and human use is higher; Figure 1). More specifically, we created two strata: the first included all ‘on‐trail’ areas along official trails and routes in the park, as well as the lodge access road, while the second included off‐trail areas defined as being greater than 350 m from these trails but within 2 km to allow reasonable access on foot. Our scope of inference therefore does not extend throughout the entire park, as we did not sample the least accessible areas, which may be used differently by wildlife. We used the random point tool in ESRI ArcMap 10.7 (ESRI, Redlands, USA) to generate points within these strata, attempting to sample roughly equal numbers of points on‐ and off‐trail (final sample sizes were *n* = 21 and 24, respectively, Figure 1). We attempted to sample similar landscapes on‐ and off‐trail in terms of features such as vegetation, habitat attributes and microtopography (as shown by the mean and range of each predictor in Table 2), with the only differences being the trail itself and associated human presence. Random points were also constrained to be a minimum of 750 m away from all other points to increase spatial independence among samples. Field teams navigated to each random point using coordinates on handheld GPS units and deployed the camera as close to the point as possible given logistical constraints encountered, such as topography and dense brush. Cameras were set facing an opening or feature (e.g. game trail) expected to maximise detections of animals using that random location.

**TABLE 2:** Predictor variables used in Bayesian regression models of wildlife detections at 45 sites in Cathedral Provincial Park, including mean and range for each predictor at on‐ and off‐trail sites.

Variable	Description	Category	On‐trail mean (range)	Off‐trail mean (range)
Humans	No. of human detection events per week at each camera site	Human use	12.13 (0–244)	0.049 (0–2)
Distance to linear^a^	Distance from camera to nearest trail or road (m) – with exponential decay	Human use	0	460 (117–1435)
Elevation^b^	Elevation at site (m)	Environmental	1821 (819–2261)	1675 (1080–2400)
VRM^c^	Site ruggedness within 90 m of the camera	Environmental	0.0018 (0.00004–0.013)	0.0028 (0.00007–0.019)
Open_500_ ^d^	% of non‐forested habitat within 500 m radius	Environmental	30 (4–99)	36 (1–99)
NDVI^e^	Normalized Difference Vegetation Index	Environmental	0.45 (0.0038–0.94)	0.46 (0.0036–0.98)

^a^ Trail features from BC Parks and OpenStreetMap.

^b^ Field Measurement.

^c^ Vector Ruggedness Measure. Formula from Sappington et al. (2007).

^d^ https://www2.gov.bc.ca/gov/content/industry/forestry/managing‐our‐forest‐resources/forest‐inventory/data‐management‐and‐access.

^e^ From MODIS MOD13Q1. https://modis.gsfc.nasa.gov.

Cameras were placed on trees with a minimum diameter > 10 cm, or a large boulder in one case above the treeline, at a height of 0.3–1.0 m above the ground. Camera sets were dependent on site characteristics and distance from the focal feature to provide a consistent detection zone for target animals. Focal features included human trails, game trails and gravel roads. Similar detectability at each camera was confirmed with the camera walk test feature by field personnel moving through the detection zone at different distances and heights (e.g. standing, crawling). At on‐trail sites, cameras were placed perpendicular to the trail wherever possible to standardise detection probability across locations. Cameras were affixed 3–6 m from the focal target zone of anticipated animal or human travel (e.g. trail). Cameras were set to take one image per trigger, with no delay between successive images and high trigger sensitivity. Once deployed, camera traps were checked every 2 months between June and October for those in the higher use core area or other accessible areas and annually for those in remote areas with limited human access. Camera operability was high, resulting in over 26,000 camera‐days of data collected and a mean of 578 trap nights per camera (median: 616, range: 91–819). The few cases of camera failure were the result of wildlife damage, hardware malfunction, human tampering, or heavy snowfall.

We limited this study to medium‐ and large‐bodied mammals, as the decreased detectability of smaller species on camera traps set to capture multiple species decreases the utility of this method for making unbiased inference about smaller animals (Kolowski & Forrester, 2017).

### 2.3 Data processing

Following the collection of data from the field, images were blurred using an artificial intelligence (AI) algorithm to protect privacy by preventing the identification of individual humans in camera trap images while still allowing the counting of human recreationists (Fennell et al., 2022). Images were then renamed and uploaded to a classification database in the WildCo lab at UBC (https://wildlife.forestry.ubc.ca). We additionally processed data through MegaDetector, a machine learning object detection model to identify humans, animals, vehicles and blank images (Beery et al., 2019; Microsoft, 2020). This step increases classification efficiency, particularly by allowing automated detection and filtering of blank and human images (Fennell et al., 2022).

The project team classified animals in each image according to species, sex (where possible, e.g. mountain goats, deer and moose) and age class (adults, juveniles and sub‐adults where possible). We also tabulated a group count of the total number of distinguishable individuals within a sequence of related detections, with a sequence (hereafter called an independent event) defined as all images of the same species occurring within 5 min of the last image of that given species. Images of commonly confused species (e.g., lynx and bobcat) were each reviewed by author MF a second time to ensure misclassification rates were minimised. Where there was uncertainty around the species within an image, an identification of ‘Unknown species’ was assigned.

Due to the immense number of human images captured, we used MegaDetector to automatically detect any images containing humans. Using a confidence threshold of 90% and manual visual confirmation, we classified all photos containing at least one human by activity type. Types of human activity included hiking, horseback riding, mountain biking and motorised vehicles; however, the latter only occurred at two sites on the private lodge access road, and horseback riding was only infrequently detected on a single trail skirting the edge of the park. Given the dominance of hiking and minimal detections of other human activities in the park, we constrained our analyses of human activity to focus only on hiking.

### 2.4 Statistical analysis

We used a Bayesian generalised linear mixed‐modelling framework to evaluate spatiotemporal responses in site use by wildlife at the weekly temporal scale. Once images were classified, the number of independent detections per week at each site was summed for each of the focal species (Table 2), serving as an index of site or habitat use through space and time (i.e., assumed to reflect species use of local resources and conditions around camera trap sites; Tattersall et al., 2020). We defined a week as the period from Wednesday to Tuesday to overlap each weekend (including holidays) centrally within each weekly sample, as the weekend is typically the time of increased visitation to the park (Nix et al., 2018). Weekly counts permit evaluation of temporal trends within and across sites and represent a balance between finer‐scale temporal units, such as daily counts, that may be dominated by no detections (Naidoo & Burton, 2020), and coarse temporal units, such as months, that may not sufficiently capture the large seasonal changes in human recreation within Cathedral when access is concentrated in the snow‐free periods between June and October. Future work could further evaluate different spatiotemporal resolutions. As human use is limited outside of these months, we interpret results in relation to potential seasonal effects, which we attempted to account for via a continuous measure of vegetative productivity (NDVI; Table 2).

The count of detections per site week represents a combination of the number of individuals of a species using that site and their movement patterns relative to the camera. We assumed that animals moving through the camera detection zone were detected with high probability, although some individuals using the surrounding area may not have passed through the detection zone. Nevertheless, we were interested in the variation in detections as a signal of variation in site use rather than sampling error as is assumed in some modelling frameworks (e.g. occupancy). Only weeks with seven active camera days were included. We have no reason to expect unmodeled bias in detections and assumed they were proportional to site use. Further, occupancy may be more sensitive to variations in movement (including seasonally) than detection‐based indices (Neilson et al., 2018; Stewart et al., 2018).

To test the hypothesis that human recreation is a major determinant of wildlife habitat use, we derived camera trap indices of human use to provide a direct signal of recreational pressure. We calculated the number of independent human detection events, defined by a 5‐min independence threshold, per active week at each camera site as our primary predictor. We also accounted for several environmental and sampling variables that could influence animal occurrence and detection at camera sites (Table 2). We included distance from camera sites to trails or roads (as an alternative but less direct measure of human disturbance, as well as a proxy for ease of animal travel), as well as elevation and terrain ruggedness (vector ruggedness measure, Sappington et al., 2007), which may influence perceived or real predation risk, or ease of movement. We included percent open habitat (<25% crown closure, from the provincial forest inventory) within 500 m of the site to represent the level of concealment available to wildlife. We also included the Normalized Difference Vegetation Index (NDVI) as a proxy for vegetative productivity, which may influence forage and, by association, prey availability for carnivores (Merkle et al., 2016). NDVI also represents seasonal changes throughout the study area, which may influence habitat use unrelated to human influence. We calculated the distance to linear features with an exponential decay, such that $d=1-e^{-1\left(\frac{\text{distance}}{500}\right)}$, as we predicted that any influence of these linear features would quickly decrease with distance. NDVI was obtained from the MOD13Q1 product using the *MODISTools* R package (Tuck et al., 2014) at a 16‐day interval within a 500 m buffer around each camera, resulting in consecutive weeks having the same value.

To parse the effects of recreation, environment or a combination of these predictors we created three candidate models for each species and conducted model selection to discern the best‐supported set of predictors (Table 2). The three models for each species included:

1. Human detections and distance to linear features (human model).
2. The environmental covariates described above without human detections or linear features (environment model).
3. All predictors (combined model, human model + environmental model).

We modelled the number of independent detections of each species per site week as a negative binomial response and included the camera site as a random intercept to account for potential nonindependence among repeated observations at the same site. All independent variables were standardised by subtracting the mean and dividing by one standard deviation to allow direct comparison of the direction and magnitude of effects on species use of habitat (Gelman, 2008). All predictor variables were assessed for collinearity (none were highly correlated, |*r*| < .5; Dormann et al., 2013) and tested for multicollinearity using the Variance Inflation Factor (VIF; all had VIF < 2).

We used Leave One Out (LOO) cross‐validation for model selection by calculating the Leave One Out Information Criterion (LOOIC) for each candidate model (Vehtari et al., 2017). We selected the model with the lowest LOOIC as the most supported, with a threshold of eight ΔLOOIC signifying a large difference between models' predictive power (Sivula et al., 2022). All species other than wolves had the combined model as the most supported. For wolves, the combined model was not the most supported yet was within eight ΔLOOIC, thus we report the results of the combined model. For full model selection results, including Bayesian *R* ^2^ values (Gelman et al., 2019), see Table S1.

We ran models in R 4.1.0 (R Core Team, 2020) using the package *brms* (Bürkner, 2017). Models ran with default non‐informative priors (uniform distribution, −∞ to ∞) for 5000 iterations on each of 4 chains with a thin rate of 1, following a burn‐in of 2500 iterations. Model convergence was assessed through inspection of trace plots and the Gelman‐Rubin statistic (Rhat <1.1; Gelman & Hill, 2007). All models were run with the full set of predictors (Table 2). We interpreted parameter estimates with Bayesian 95% credible intervals drawn from the posterior that do not include zero as signifying strong evidence of an effect of that variable in influencing species' use of sites, and Bayesian 80% credible intervals not overlapping zero as signifying weak evidence of an effect. We assessed effective sampling in the model by calculating the number of effective samples against the number of total samples (*n*_eff_ */N* > 0.1; Geyer, 1992).

In addition to estimating the spatial effects of recreation on wildlife at the weekly scale, we evaluated finer‐scale temporal interactions by analysing changes in diel activity for each species. This method allows inferences about the fine‐scale temporal displacement of species by analysing the proportion of detections across a 24‐h period in radian time (Frey et al., 2020; Rowcliffe et al., 2014). We calculated the coefficient of overlap (Δ; Ridout & Linkie, 2009; Schmid & Schmidt, 2006) between all independent detections of each species and humans, as well as between the activity of each species at on‐ vs. off‐trail sites. The coefficient of overlap quantifies the overlap of two activity patterns and ranges from zero to one, with values closer to one signifying higher overlap. Using the *overlap* R package (Meredith & Ridout, 2009), we generated 95% confidence intervals for each overlap estimate using 10,000 bootstraps and presented visual representations of each activity curve on a 24‐h axis. We used Δ_4_ in cases with ≥50 observations in each group and Δ_1_ in cases with <50 observations per group (Table 3; Meredith & Ridout, 2009; Ridout & Linkie, 2009). To further quantify the difference between activity for on‐ and off‐trail sites, as well as between each species and humans, we generated a kernel density estimate from each activity distribution, using 10,000 bootstrap samples from the data for each, using the *activity* R package (Rowcliffe et al., 2014). We then conducted a Wald test to compare the difference between these kernel density estimates, testing the null hypothesis of no difference between groups and with *p* values <.05 representing statistically significant differences between activity patterns.

**TABLE 3:** Number of independent detection events, as well as on‐ and off‐trail detections and the ratio of on‐ to off‐trail detections for each species of interest in Cathedral Provincial Park.

Species	Total detections	On‐trail detections	Off‐trail detections	On‐trail:Off‐trail
Humans	21,646	21,543	103	209.16
Mule deer	2186	1333	853	1.56
Coyote	593	510	83	6.14
Lynx	419	401	18	22.28
Mountain goat	333	257	76	3.38
Black bear	171	94	77	1.22
Moose	128	58	70	0.83
Wolf	78	66	12	5.50
Cougar	38	34	4	8.50
Total Animals	3830	2753	1193	2.31

*Note*: Off‐trail detections of humans include the study team.

## 3 RESULTS

Our most frequently detected wildlife species of interest was mule deer (*n* = 2186 independent events), followed by coyote (*n* = 593) and lynx (*n* = 419) (Table 3). We recorded 21,646 human detections, as well as 154 domestic dog detections (generally alongside humans), despite dogs being banned in the park.

### 3.1 Spatial effect

**FIGURE 2:**
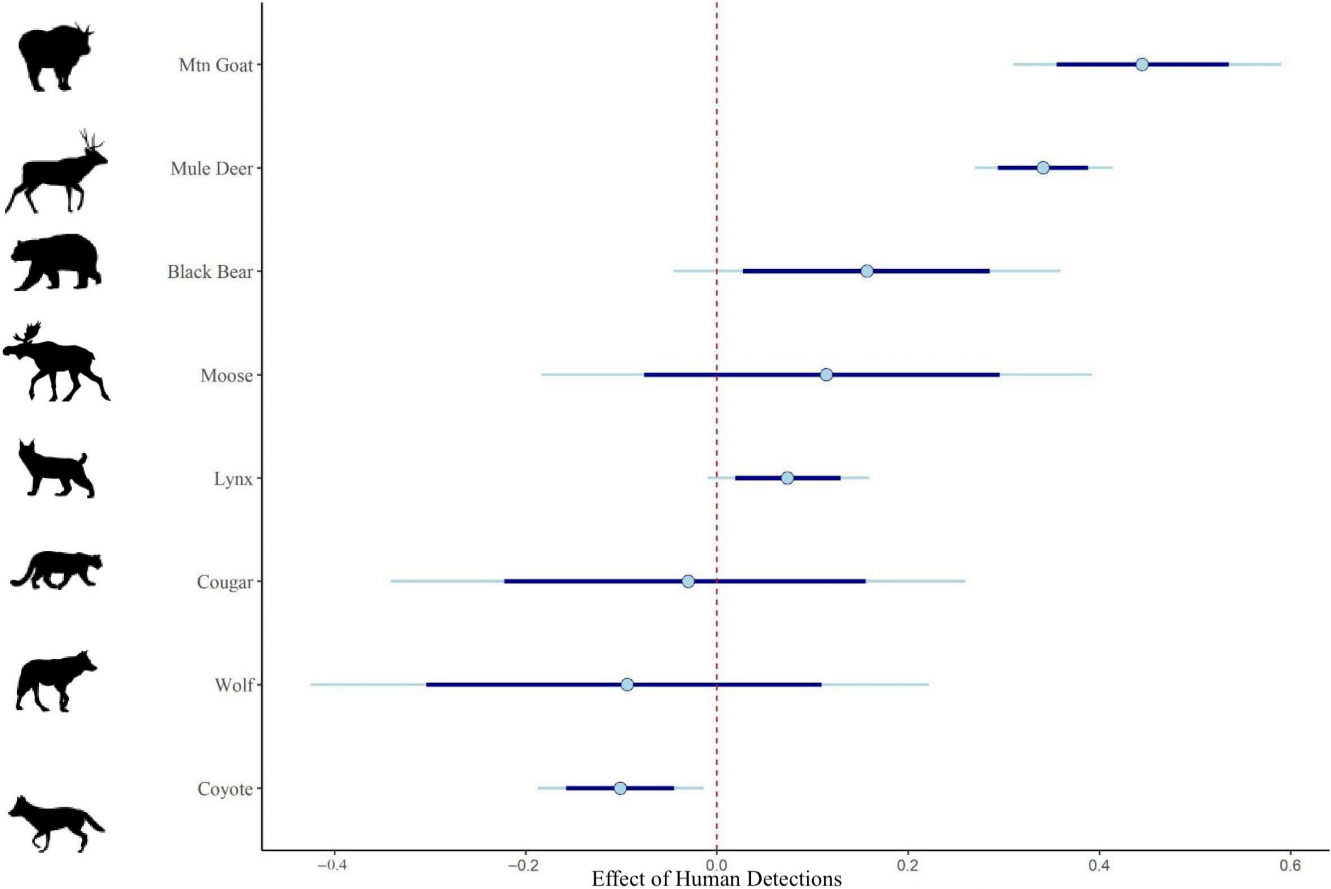
Effect of human detections on species habitat use. Shown are posterior mean parameter estimates and credible intervals from Bayesian GLMMs with all predictor variables using detections at camera trap sampling sites at a weekly scale (see Table 2 for the full set of parameters included in models). Thick lines represent 80% credible intervals, and thin lines represent 95% credible intervals. Values to the left of zero (vertical dashed line) suggest avoidance of areas with more human detections, while values to the right of zero suggest attraction to areas with more human detections.

**FIGURE 3:**
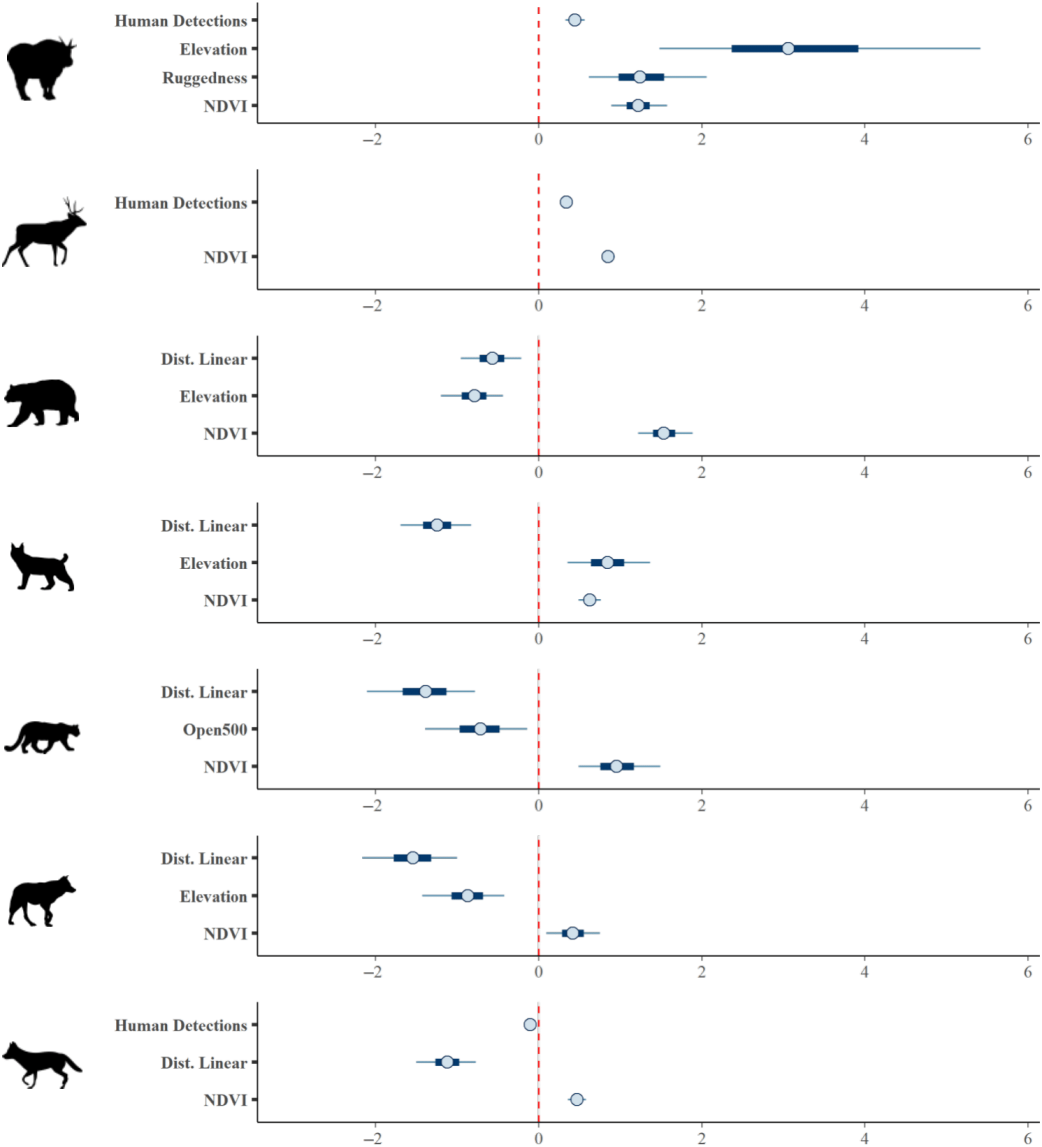
Parameter estimates for strong predictors of wildlife habitat use. Shown are parameter estimates from Bayesian GLMMs. Strong predictors are defined as those for which the 95% credible interval does not overlap zero. Species are (top to bottom): mountain goat, mule deer, black bear, Canada lynx, cougar, wolf and coyote. Thick lines represent 80% credible intervals, and thin lines represent 95% credible intervals. Credible intervals are not visible for some estimates at this scale. All predictors were standardised to have a mean of 0 and standard deviation of 1. Moose are not shown since the 95% intervals for all predictors overlapped zero.

We found strong evidence that human recreation affected habitat use by mountain goats and mule deer, with a positive relationship between the number of human detections per site‐week and the number of detections of these species (Figures 2 and 3). Coyote was the only species where recreation had a strong negative effect on habitat use (Figures 2 and 3). We saw weak evidence that black bear and lynx had more detections where human use was higher, while we found no evidence that recreation influenced spatial patterns of habitat use by moose, cougar, or wolf (Figure 2). Detections of all carnivore species were negatively correlated with the distance to the nearest linear features, suggesting that predators were more frequently detected closer to recreational features such as roads or trails (Figure 3).

In terms of species associations with environmental features, wolf and black bear were detected more often at lower elevations, while mountain goat and lynx were detected more often at higher elevations (Figure 3). The effect of terrain ruggedness was significant and positive only for mountain goat habitat use, and the percent open habitat around a camera was significantly negatively correlated with cougar occurrence (Figure 3). NDVI was significantly positively correlated with all species other than moose (Figure 3). Full model results for each species are available in Tables S2–S9.

### 3.2 Temporal partitioning

**FIGURE 4:**
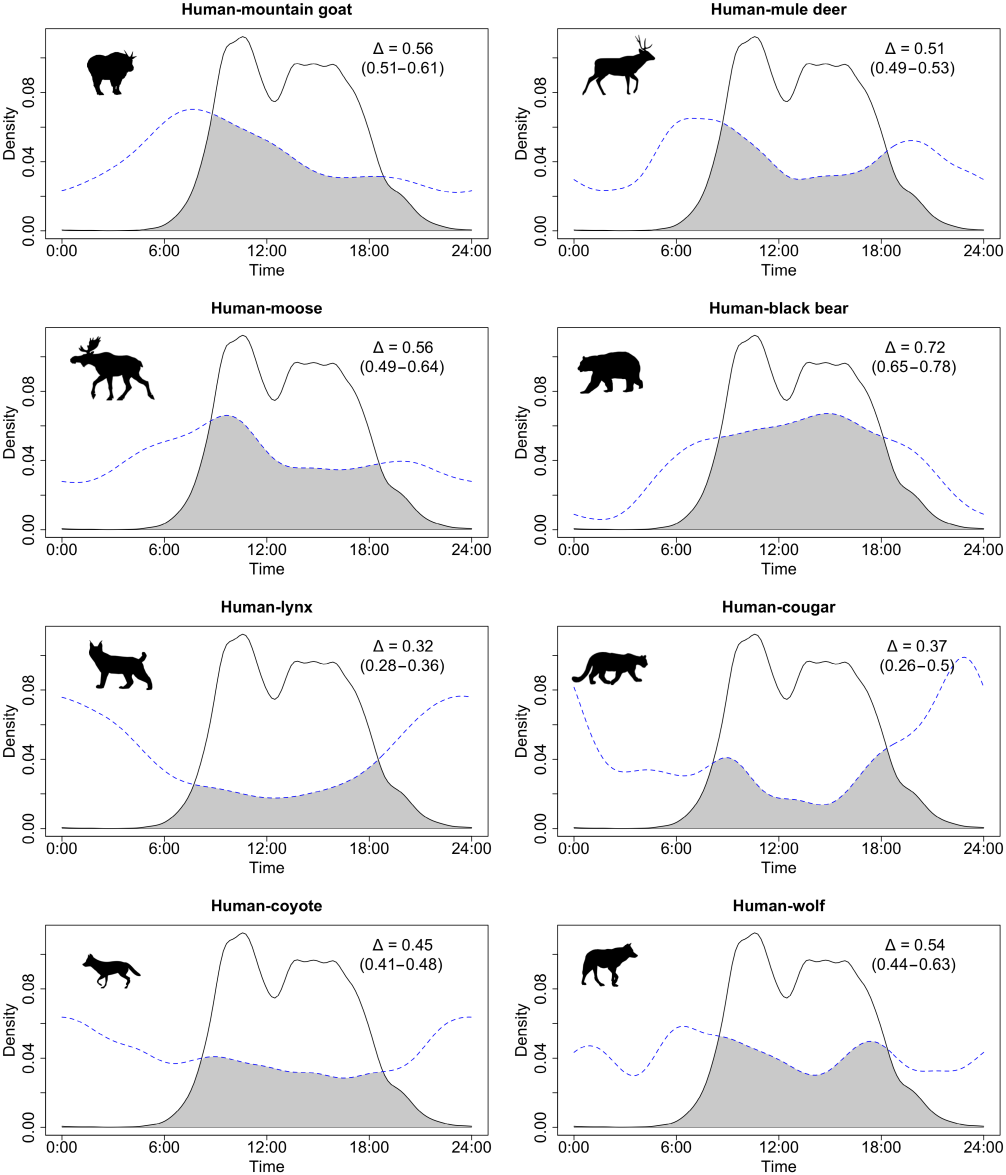
Human (solid line) and animal (dashed line) comparisons of diel activity measured by camera trap detections for eight focal species, with overlap coefficient (Δ) estimates (generated with 10,000 bootstraps).

**FIGURE 5:**
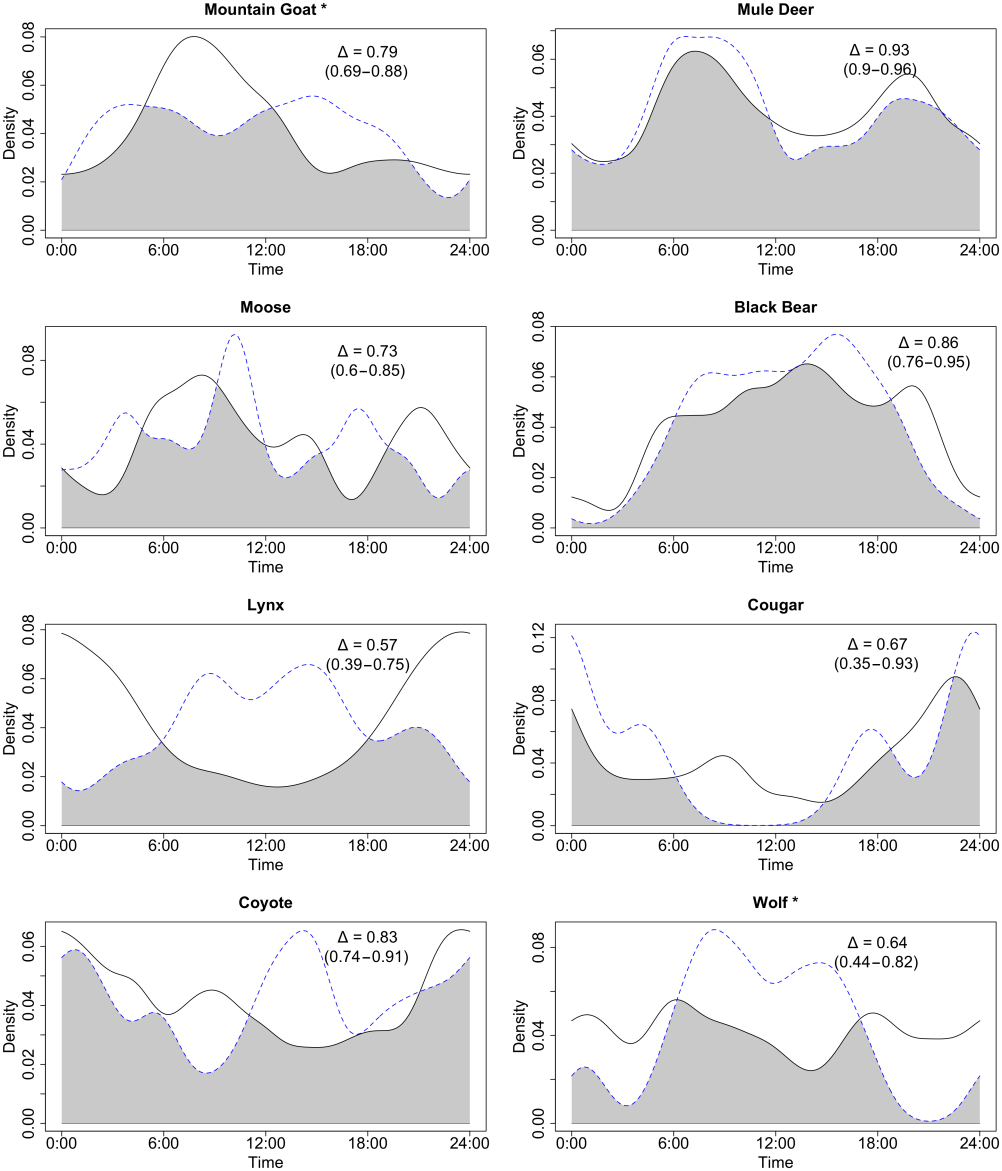
Comparisons of diel activity for each species between camera trap sites on (solid line) vs. off (dashed line) of recreation trails, with overlap coefficient (Δ) estimates (generated with 10,000 bootstraps). Note the varied *y*‐axis scale between each plot, which represents the kernel density estimate. Asterisks next to species names represent significant Wald test results between on‐ and off‐trail activity curves.

All species showed relatively low temporal overlap with humans in their diel activity patterns, supporting our prediction of temporal partitioning from people for large carnivores and mesocarnivores but contrary to our prediction of higher temporal overlap between ungulates and people (Figure 4, Table 4). Cougar activity patterns were not significantly different from humans, though this was potentially due to a few cougar detections (Figure 4, Table 4). Most predator species were more nocturnal than humans, except for black bears which, like people, exhibited a diurnal activity pattern. Ungulates were generally more crepuscular than predators or humans. Mountain goats and wolves were the only two species with significant differences in their diel activity patterns between on‐ and off‐trail sites (Figure 5, Table 4). Mountain goat activity occurred earlier in the day at on‐trail sites in comparison to off‐trail sites (Wald test, *W* = 6.85, df = 1, *p* = .0089), and wolves used on‐trail sites significantly more at night than during the day (Wald test, *W* = 4.36, df = 1, *p* = .037).

**TABLE 4:** Overlap coefficient (with 95% confidence interval as generated by 10,000 bootstrap samples), Wald statistic and Wald test *p*‐value for activity patterns of single species on‐ versus off‐trail and human versus each species.

Species	On vs. off‐trail			Overlap with humans		
	Δ (95% CI)	*W*	*p*	Δ (95% CI)	*W*	*p*
Mountain goat	0.79 (0.69–0.88)	**6.85**	**.0089**	0.56 (0.51–0.61)	**22.09**	**<.0001**
Mule deer	0.93 (0.90–0.96)	1.15	.2842	0.51 (0.49–0.53)	**109.05**	**<.0001**
Moose	0.73 (0.60–0.85)	0.93	.3360	0.56 (0.49–0.64)	**8.71**	**.0032**
Black bear	0.86 (0.76–0.95)	1.01	.3155	0.72 (0.65–0.78)	**19.99**	**<.0001**
Lynx	0.57 (0.39–0.75)	0.81	.3685	0.32 (0.28–0.36)	**23.19**	**<.0001**
Cougar	0.67 (0.35–0.93)	0.41	.5202	0.37 (0.26–0.50)	0.24	.6218
Coyote	0.83 (0.74–0.91)	<0.01	.9808	0.45 (0.41–0.48)	**31.03**	**<.0001**
Wolf	0.64 (0.44–0.82)	**4.36**	**.0368**	0.54 (0.44–0.63)	**14.55**	**.0001**

*Note*: Statistically significant differences between activity patterns are denoted in bold.

## 4 DISCUSSION

Our results are consistent with the hypothesis that human recreation may impact the timing and location of activity by wildlife within a protected area. However, the observed shifts in space and time were highly variable across species. We found that only coyotes displayed patterns of habitat use consistent with strong spatial displacement from recreation on a weekly scale. For two of the three ungulate species, our prediction of a positive spatiotemporal correlation with human recreation was supported, providing partial evidence for the human shield hypothesis, though our prediction that predators would avoid areas of higher human use was not strongly supported (Muhly et al., 2011). Our predictions of displacement or neutral temporal responses to recreation for mountain goats and mule deer, respectively, were not supported, and we suggest that behavioural plasticity (i.e. ability to adjust to stressors) may allow these species to spatially co‐occur with human recreation (George & Crooks, 2006).

Mountain goats significantly shifted diel activity away from times of recreation activity and were one of two species to show significantly different activity patterns between on‐ and off‐trail sites, suggesting a high level of plasticity in response to human disturbance. We suggest that this temporal shift may result from goats attempting to balance the negative effects of direct human interaction during the day with the positive nutritional benefits of human‐derived salt sources (such as urine, grey water or sweaty clothing) at night (Kroesen et al., 2020; Sarmento & Berger, 2017). Mule deer exhibited crepuscular activity patterns with minimal changes in activity pattern between on‐ and off‐trail sites, which is consistent with the findings of Reilly et al. (2017).

Larger predator species did not generally show strong responses to recreation, contrasting our prediction that they would avoid people. Black bears were weakly positively correlated with the number of weekly human detections, suggesting that this species shares space with humans, contrary to our predictions under the human shield hypothesis, though use of habitat near humans may be influenced by easier travel by predators on trails and roads (Dickie et al., 2017). Black bears may also be attracted to food sources associated with human activity (e.g., garbage, camp food; Klees van Bommel et al., 2022). Other potential reasons for the observed lack of expected predator avoidance of people could include low hunting pressure (i.e., low risk of mortality associated with human encounters) and relatively low predator population density, decreasing inter‐ or intraspecific competition for habitat (as well as reducing sample sizes and thus statistical power to characterise responses). Wolves and cougars were the least frequently detected focal species and did show negative mean estimates for the recreation effect but with wide confidence intervals (Figure 4), suggesting further study is warranted. Wolves were also the only predator to show significantly greater use of on‐trail sites at night, suggesting they may have been avoiding trails when they were used by people.

Mesopredator spatiotemporal responses to human recreation were mixed. Consistent with our prediction under the human shield hypothesis (Table 1), the occurrence of lynx was positively correlated with human activity (though weakly), while contrary to our prediction, coyotes were strongly negatively correlated with recreation. Lynx and coyotes each had significantly different diel activity patterns from humans, with both being more active at night, consistent with results from prior studies (Nickel et al., 2020; Reilly et al., 2017), which have shown increased cathemeral (irregular throughout day and night) activity by coyotes in areas of co‐occurrence with wolves, potentially due to competition or intraguild predation (Frey et al., 2020; Shores et al., 2019). While we did not statistically test for such an interaction, it may explain the observed difference in on‐ and off‐trail diel patterns between these canid species. Additionally, we did not directly investigate patterns of occurrence for predominant mesocarnivore prey (squirrels, hares and other small mammals) due to their small body size, resulting in inconsistent detectability on camera traps. Therefore, the mechanisms underlying mesocarnivore responses to human activity remain to be further tested.

Increased use of trails at night by mountain goats and wolves suggests that these linear features may play an important role as travel corridors while potentially facilitating resource acquisition. For mountain goats, trails may allow faster travel to natural mineral licks while also providing a direct source of salt due to trailside urination by humans, as well as leading to campgrounds that may serve as artificial mineral licks due to concentrated human use (Kroesen et al., 2020; Sarmento & Berger, 2017). Predators, particularly wolves, use linear features to increase hunting efficiency in other environments (Dickie et al., 2017). While wolves may not be able to use these corridors during the daylight hours when they are dominated by human recreationists, trails may directly result in increased exploitation of prey species by these predators at night, resulting in indirect negative effects of recreation infrastructure on prey. This effect is further supported by the negative correlation between detections of all predator species and distance to linear features, suggesting that the five carnivore species are frequently close to roads or trails. The interplay between predator attraction to trails and repulsion from people is an interesting avenue for future research.

Expansion of the ‘human niche’ in terms of habitat use in space and time results in species‐specific responses by wildlife (Gilbert et al., 2022). Although we did not specifically investigate the consequences of observed behavioural responses for individual fitness or population demography, prior research has shown effects ranging from decreased time for foraging (Coppes et al., 2017) or hunting (Musiani et al., 2010), to reduced fecundity (Phillips & Alldredge, 2000), to wholesale abandonment of territories (Pauli et al., 2017). Despite the potential for positive impacts on ungulate survival under the human shield hypothesis, human alteration of natural food webs via recreation is contrary to the mandate of many protected areas to preserve and protect natural environments. The plasticity of animal responses to human disturbance provides the prospect for landscapes of coexistence, though thresholds of sensitivity likely vary by species (Kronfeld‐Schor & Dayan, 2003; Oriol‐Cotterill et al., 2015). Sensitive species might be more readily lost from landscapes facing human pressures, presenting a form of ecological filtering of the wildlife community. Examples of species particularly sensitive to disturbance that have historically been present in this study area yet are rarely detected and potentially extirpated include wolverine (*Gulo gulo*; Stewart et al., 2016) and grizzly bear (*Ursus arctos*; Sarmento & Berger, 2017). Cathedral provides an example of a relatively low‐use protected area in comparison to more commonly visited areas in North America, such as Banff or Yellowstone National Parks, though its small size, isolation and alpine environment may increase sensitivity to cumulative effects, including recreation. It is important to note that our study sampled only a portion of the total area of Cathedral, covering a gradient from the high‐use core area to areas of less intensive human use, seeing only a handful of recreationists each year. Due to logistical constraints, we were not able to sample the entire protected area, including the areas most distant from human use. Only by extending our sampling gradient to include these more remote areas could we determine if they are used by more sensitive species and if the relationships between wildlife and recreation that we estimated apply throughout the entire park. Particularly of interest for future study are the ‘natural’ temporal patterns of each species when away from human influence.

Protected areas assist in maintaining mammal diversity (Chen et al., 2022), reduce population declines (Geldmann et al., 2013) and are potential refugia from climate change (Haight & Hammill, 2020). Using the best available science to inform management decisions serves to ensure that protected areas will continue to provide these benefits to wildlife in the future while also ensuring opportunities for human enjoyment of these landscapes. Translating research into effective management requires collaboration between researchers and practitioners (Lemieux et al., 2018; Merkle et al., 2019). Here, we provide insight on the relationships between recreation and a range of wildlife species within one protected area facing pressures from increasing recreation, decreasing landscape connectivity and looming climate change impacts, providing results that may be used by park managers to inform actions such as managing the location, timing or intensity of recreation activities. Possible actions might include spatial or temporal closures of trails or areas within protected areas, permitting systems that maintain recreation under a designated threshold, or the intentional creation of trail networks away from important habitat features for sensitive species. Our results are specific to the current levels of recreation in Cathedral, and may thus serve as a baseline in the face of increased recreation over time and as a comparison with other systems facing different levels of recreation. Further, our analyses depict patterns of habitat use by each species but do not account for factors such as population abundance or demography, which are important to consider for effective landscape‐scale management, and our assessment was constrained to more accessible portions of the park near trails, providing an incomplete picture of wildlife use of the entire protected landscape. Additionally, many species may make seasonal movements throughout the study area naturally, which we did not directly account for in our modelling framework, although exploration of seasonal effects may be an exciting future direction for research (Ager et al., 2003; Aikens et al., 2017; Merkle et al., 2016).

While we show statistically significant relationships between recreation and habitat use for a number of species, we do not quantify what the physiological or population effects of these responses may be. Prior research has suggested impacts from perceived risk (whether from predators or humans) may include reduced fecundity or decreased body condition, leading to negative effects individually and for the population (Clinchy et al., 2013; Creel et al., 2009; Phillips & Alldredge, 2000). Additional studies at different scales may further inform understanding of anthropogenic impacts on sensitive wildlife, such as a concurrent study in Cathedral that used GPS collars to assess the impacts of helicopters on mountain goats (Balyx, 2022). Further, our study investigated relationships between non‐consumptive recreation and wildlife but did not account for consumptive harvest (hunting), which also occurs in limited amounts within Cathedral. Managers should consider existing policies for both consumptive and non‐consumptive recreation when interpreting these results, and integrating the cumulative effects of multiple types of impact is an important avenue for future research and application.

## 5 CONCLUSION

Understanding animal responses to recreation is not only important for advancing animal ecology; it is critical to the effective management of protected areas and other wildlife habitats. Protected areas in particular frequently face a dual mandate dilemma, where balancing ecological integrity with recreational opportunities requires a thorough understanding of the potential impacts of different management decisions. Here, we found relatively limited spatial displacement of wildlife by recreation but documented altered spatial and temporal patterns of habitat use across several species. This information can be used by managers when planning to balance the dual mandate, such as through the delineation of sensitive times when wildlife is more active and associated restrictions on the timing of visitor use. Our study highlights the importance of monitoring both human and wildlife activity to improve understanding of recreation impacts, and we recommend the implementation of long‐term monitoring programs. Coexisting in both space and time is crucial for people and animals to thrive in these natural spaces, and promoting such coexistence requires an understanding of animal responses to all human activities, even ‘low impact’ activities like hiking.

While coexistence between wildlife and recreationists is clearly possible, land managers will increasingly have to walk a fine line between protecting habitats for wildlife and making those habitats available for recreation. Despite a history of ‘fortress conservation’ that attempted to largely exclude humans from protected areas (Saarinen, 2016), there is a much longer history of coexistence between people and nature in landscapes stewarded by Indigenous peoples in North America and elsewhere. Moving forward, models of conservation that embrace such coexistence are needed and can be seen in emerging examples such as Indigenous Protected and Conserved Areas (IPCA's), which inherently include humans as both stewards and users of the land and acknowledge that the evolution of much of the landscape across Canada has been influenced by humans for millennia (Moola & Roth, 2019). One such IPCA has recently been declared in the landscape that includes Cathedral Park, affording a new avenue for planning and adaptive management of recreation and other pressures in this area (Lower Similkameen Indian Band, 2022). These more integrated operational frameworks for protected areas, in combination with a growing body of knowledge on recreation ecology, contribute to an improving landscape of coexistence for wildlife and recreationists in Cathedral and beyond.

## AUTHOR CONTRIBUTIONS

**Mitchell J. E. Fennell:** Conceptualization (equal); data curation (lead); formal analysis (lead); funding acquisition (supporting); investigation (lead); methodology (lead); project administration (equal); validation (lead); visualization (lead); writing – original draft (lead); writing – review and editing (equal). **Adam T. Ford:** Conceptualization (supporting); formal analysis (supporting); investigation (supporting); methodology (supporting); writing – original draft (supporting); writing – review and editing (equal). **Tara Martin:** Conceptualization (supporting); formal analysis (supporting); investigation (supporting); methodology (supporting); writing – original draft (supporting); writing – review and editing (equal). **A. Cole Burton:** Conceptualization (equal); data curation (supporting); formal analysis (supporting); funding acquisition (lead); investigation (supporting); methodology (supporting); project administration (equal); resources (lead); supervision (lead); writing – original draft (supporting); writing – review and editing (equal).

## CONFLICT OF INTEREST STATEMENT

The authors have no competing interests or conflicts of interest to declare.

## Supporting information


Appendix S1

## Data Availability

Data and code are available on FigShare: 10.6084/m9.figshare.24076713.v1.
