# Supplementary material for: Assessing the impacts of recreation on the spatial and temporal activity of mammals in an isolated alpine protected area
Source: Ecol Evol. 2023 Nov 28;13(11):e10733. doi: 10.1002/ece3.10733 (PMC10682857; doi:10.1002/ece3.10733)

**Assessing the Potential for Recreational Impacts on Mammal Habitat Use: A Case Study in Cathedral Provincial Park, Canada**

Mitchell J.E. Fennell^1*^, Adam T. Ford^2^, Tara G. Martin^1^, A.Cole Burton^1^

^1^ Faculty of Forestry, University of British Columbia, Vancouver BC

^2^ Irving K Barber Faculty of Science, University of British Columbia Okanagan, Kelowna BC

^*^Corresponding author: Mitchell.fennell@gmail.com

# **Supplemental Material**

**Table S1.** Model selection results for all species, using the Leave One Out Information Criterion. LOOIC values within 8 are considered similarly predictive. Bayesian R^2^ values represent the explanatory power of each candidate model.

| **Species** | **Model** | **LOOIC** | **Δ LOOIC** | **R^2^** |
| --- | --- | --- | --- | --- |
| Moose | Combined | 1030.6 | 0.0 | 0.030 |
|  | Environment | 1031.9 | 1.3 | 0.029 |
|  | Human | 1032.5 | 1.9 | 0.026 |
| Coyote | Combined | 2711.4 | 0.0 | 0.248 |
|  | Environment | 2717.4 | 6.0 | 0.244 |
|  | Human | 2764.6 | 53.2 | 0.213 |
| Wolf | Environment | 614.2 | 0.0 | 0.035 |
|  | Combined | 614.7 | 0.5 | 0.035 |
|  | Human | 620.3 | 6.1 | 0.024 |
| Lynx | Combined | 1906.2 | 0.0 | 0.236 |
|  | Environment | 1906.2 | 0.0 | 0.228 |
|  | Human | 1965.8 | 59.6 | 0.231 |
| Mule deer | Combined | 6264.1 | 0.0 | 0.397 |
|  | Environment | 6363.4 | 99.3 | 0.239 |
|  | Human | 6594.8 | 330.7 | 0.497 |
| Mountain goat | Combined | 796.1 | 0.0 | 0.626 |
|  | Environment | 834.2 | 38.1 | 0.422 |
|  | Human | 837.7 | 41.6 | 0.580 |
| Cougar | Combined | 374.1 | 0.0 | 0.029 |
|  | Environment | 382.6 | 8.5 | 0.025 |
|  | Human | 389.7 | 15.6 | 0.016 |
| Black Bear | Combined | 1144.0 | 0.0 | 0.089 |
|  | Environment | 1148.3 | 4.3 | 0.086 |
|  | Human | 1238.1 | 94.1 | 0.050 |

**Table S2.** Moose Bayesian model parameter estimates, including standard error, lower and upper 95% credible intervals. All predictors were standardized to have a mean of 0 and standard deviation of 1.


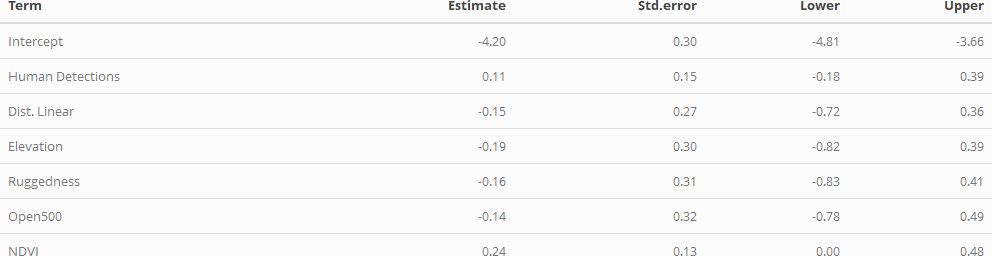


**Table S3.** Coyote Bayesian model parameter estimates, including standard error, lower and upper 95% credible intervals. All predictors were standardized to have a mean of 0 and standard deviation of 1.


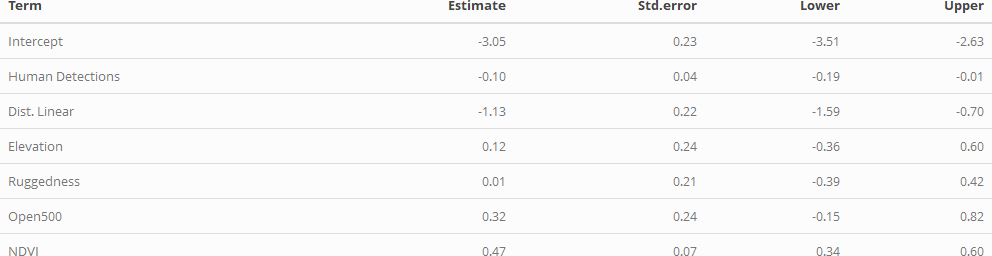


**Table S4.** Wolf Bayesian model parameter estimates, including standard error, lower and upper 95% credible intervals. All predictors were standardized to have a mean of 0 and standard deviation of 1.


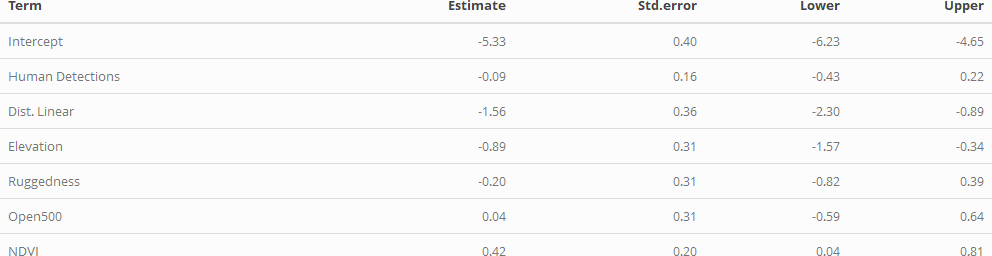


**Table S5.** Lynx Bayesian model parameter estimates, including standard error, lower and upper 95% credible intervals. All predictors were standardized to have a mean of 0 and standard deviation of 1.


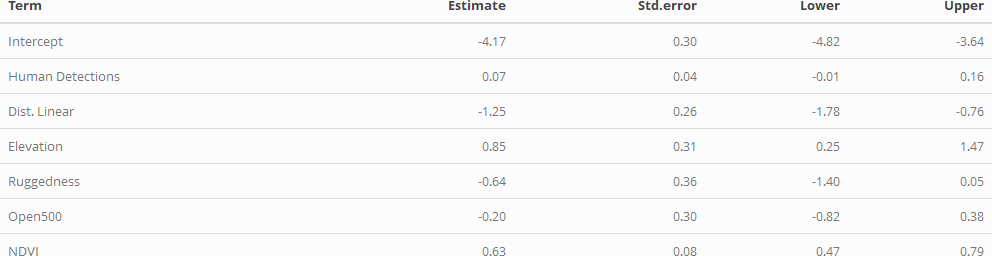


**Table S6.** Mule deer Bayesian model parameter estimates, including standard error, lower and upper 95% credible intervals. All predictors were standardized to have a mean of 0 and standard deviation of 1.


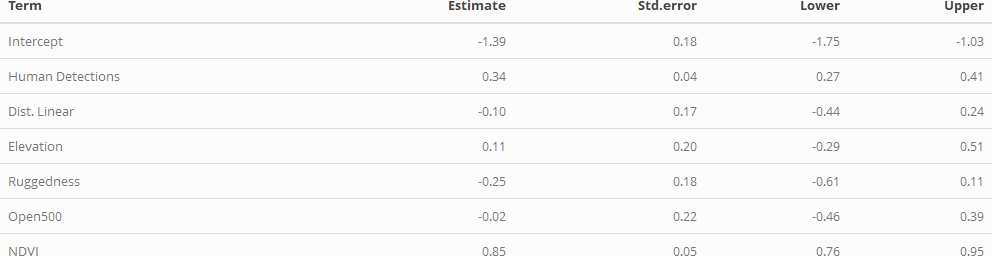


**Table S7.** Mountain goat Bayesian model parameter estimates, including standard error, lower and upper 95% credible intervals. All predictors were standardized to have a mean of 0 and standard deviation of 1.


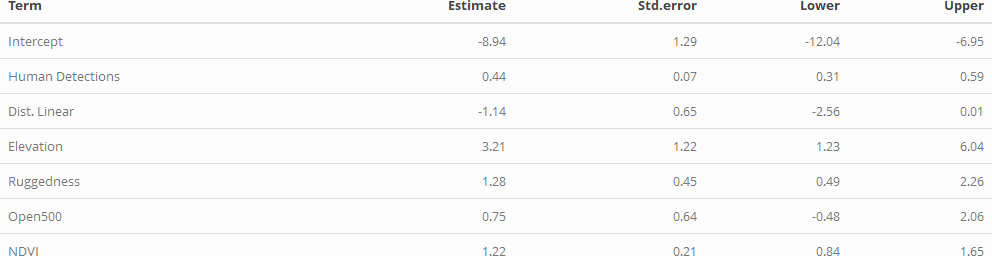


**Table S8.** Cougar Bayesian model parameter estimates, including standard error, lower and upper 95% credible intervals. All predictors were standardized to have a mean of 0 and standard deviation of 1.


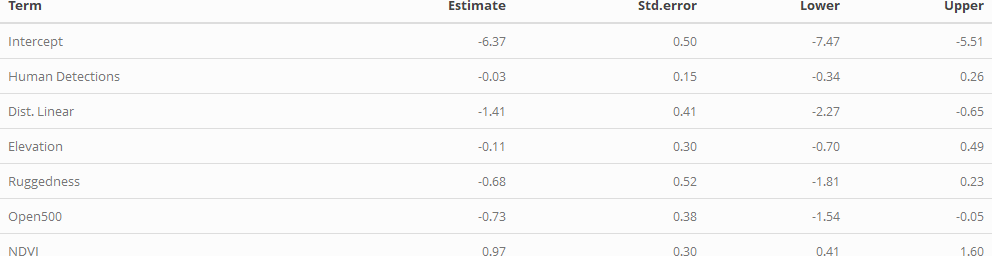


**Table S9.** Black bear Bayesian model parameter estimates, including standard error, lower and upper 95% credible intervals. All predictors were standardized to have a mean of 0 and standard deviation of 1.


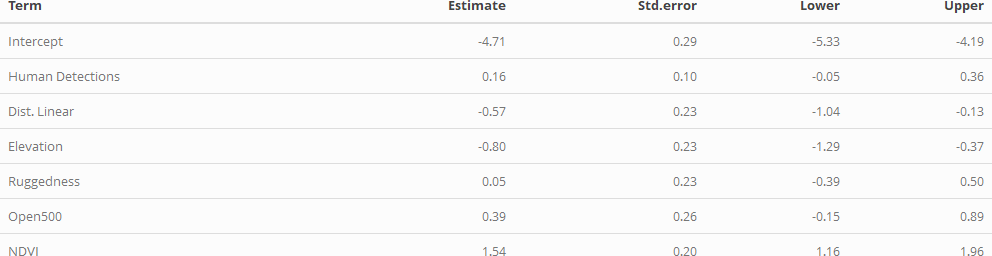

Supplement: Supplementary file 1 — Appendix S1 [file ECE3-13-e10733-s001.docx]
